# Fe doped Magnetic Nanodiamonds made by Ion Implantation

**DOI:** 10.1038/srep41938

**Published:** 2017-02-09

**Authors:** ChienHsu Chen, I. C. Cho, Hui-Shan Jian, H. Niu

**Affiliations:** 1 Nuclear Science and Technology Development Center, National Tsing Hua University, HsinChu 30013, Taiwan; 2 Department of Medical imaging and Radiological Sciences, Chang Gung University, Taoyuan 33302, Taiwan; 3 Department of Biomedical Engineering and Environment Science, National Tsing Hua University, HsinChu 30013, Taiwan

## Abstract

Here we present a simple physical method to prepare magnetic nanodiamonds (NDs) using high dose Fe ion-implantation. The Fe atoms are embedded into NDs through Fe ion-implantation and the crystal structure of NDs are recovered by thermal annealing. The results of TEM and Raman examinations indicated the crystal structure of the Fe implanted NDs is recovered completely. The SQUID-VSM measurement shows the Fe-NDs possess room temperature ferromagnetism. That means the Fe atoms are distributed inside the NDs without affecting NDs crystal structure, so the NDs can preserve the original physical and chemical properties of the NDs. In addition, the ion-implantation-introduced magnetic property might make the NDs to become suitable for variety of medical applications.

Nanoparticle mediated cancer treatments have been widely studied in recent years. The applications can be generally divided into two categories. One is for drug delivery through functionalized nanoparticles by surface modification. The other is for localized radiation therapy such as radio frequency (RF) thermal therapy and boron neutron capture therapy (BNCT)^1^,^2^. The former requires the particles having a chemically active surface so they can be functionalized to carry the needed chemicals. The later requires the particles to have certain physical properties so that they can respond to electro-magnetic (EM) waves or other radiations. No matter which application, however, biocompatibility is the foremost requirement. Nanodiamonds (NDs) have been shown to be an ideal nontoxic agent to perform such functions. Various functionalized NDs have been demonstrated to be effective in delivering drugs to cancer cells. NDs, however, are physically very inert and stable^3^,^4^,^5^. They are not magnetic to be able to respond to the EM field. They also cannot be easily excited by particle irradiation to induce nuclear reactions to be useful for localized cancer treatment. Other candidates such as nanoparticles of iron compounds, which can be easily excited by EM field to perform thermal therapy, and Boron-10 compounds, which have a very large neutron capture cross section to perform BNCT, all have shown some level of cytotoxicity, and are not perfectly biocompatible. Therefore, the future success of using Boron-10 or magnetic materials for hyperthermia or radiation therapy depends greatly on our ability to find the proper agents to carry these materials to the target of interest^6^. Since NDs are excellent drug delivery agent, it will be ideal if we can add additional physical properties so that they can also function as an agent for the radiation therapy mentioned above.

In this study, we report, for the first time, Fe doped magnetic NDs using ion implantation technique^7^. Ion implantation, a widely used application in semiconductor device fabrication, is a powerful tool for adding impurities to a host material. It has been previously reported that proton irradiation can be used to make NDs fluorescent due to defects generated by the irradiation process^8^. The damage to the crystal lattice can be easily repaired by proper post implant thermal annealing. In our study, the crystal structure of NDs is maintained while they become magnetic particles because of the added Fe atoms. Magnetic NDs can be used for many biomedical applications as a promising theranostic tool, including Magnetic Resonance Imaging (MRI) diagnostics, drug delivery and novel therapeutics. Such magnetic property in NDs can induce image contrast enhancement and enable a direct image-guided drug delivery^9^.

## Results and Discussions

**Figure 1:**
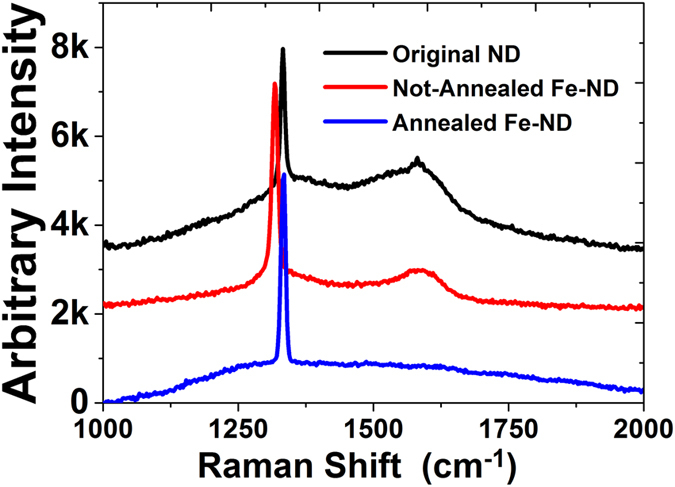
The figure shows measured Raman spectra of the original NDs, the NDs after Fe implantation and the NDs after implant and annealing. The original NDs had a diamond peak at 1332.5 cm^−1^ and a broad peak at 1579 cm^−1^, which is probably due to surface graphitic structure. After annealing, the diamond peak restored to the original position of 1332.4 cm^−1^ and the FWHM was reduced to 11.1 cm^−1^.

The ion implantation unavoidably introduces damage to the crystal lattice that receives the implantation. As mentioned above an annealing process was used to repair the damage of the NDs. Raman spectroscopy was used to examine the crystal property of NDs before and after implantation. Figure 1 shows the measured Raman spectra of the original NDs, the NDs after Fe implantation and the NDs after implant and annealing. The excitation source was a 532 nm green laser. The original NDs had a diamond peak at 1332.5 cm^−1^ and a broad peak at 1579 cm^−1^, which is probably due to surface graphitic structure. The full width at half maximum (FWHM) of the diamond peak was 10.1 cm^−1^. After Fe ion implantation, the diamond peak shifted to 1317 cm^−1^, accompanied with a much wider peak (FWHM of 98.5 cm^−1^) at 1575 cm^−1^. Due to the Fe ions are heavier in mass, when the implanted Fe ions sit in the diamond lattice (whether the one replace the carbon atom or relax in interstitial sites in diamond lattice), it will introduce strain in the nanodiamond crystal. Hence, the strain in the diamond nanoparticle roots for redshift in the Raman spectrum. The red shift of the Raman peak and the widening of the FWHM indicate clearly that the crystal lattice was damaged. However after annealing, the diamond peak became very strong and was restored to the original position of 1332.4 cm^−1^ and the FWHM was reduced to 11.1 cm^−1^. The other peak related to the surface sp2 bonds, however, has disappeared. This clearly indicates that the diamond structure is restored and the unwanted surface graphitic structure or its compounds were cleared up. In this study, the goal is to show that the Fe implanted NDs can maintain its original NDs properties. From the Raman measurement, the measurement result implies the goal was indeed achieved - although the structure was damaged by ion implantation, the outer layer can be repaired by the post annealing.

**Figure 2:**
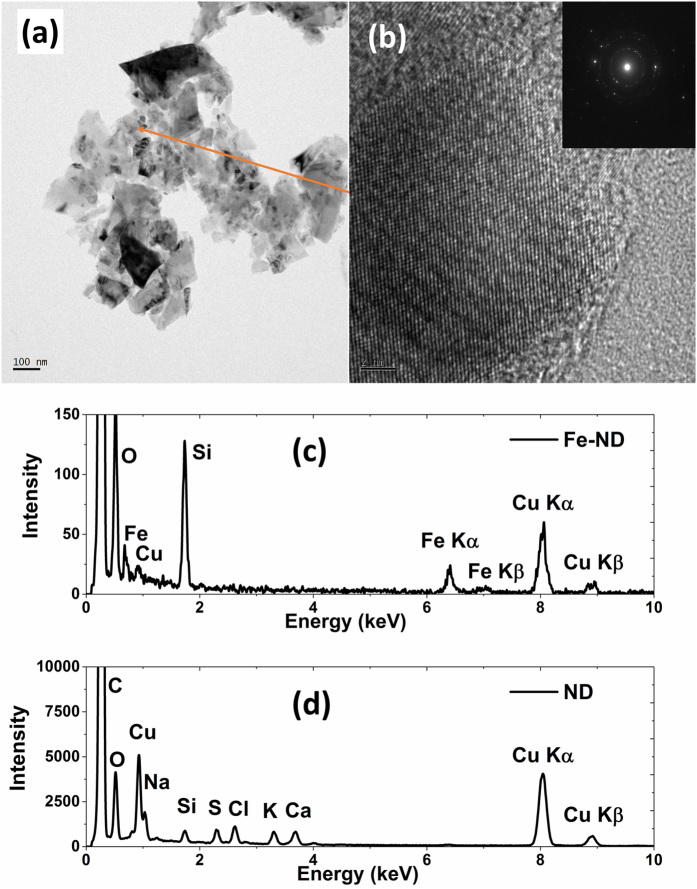
The aggregated NDs image is shown in (**a**) and that of a high resolution lattice image of a single ND is shown in (**b**). The spectrum of the Fe-implanted ND with the image shown in (**b**) is shown in (**c**) and the Fe content in the ND was estimated around 0.2% in weight by EDX. The EDX spectrum of an original ND is shown in (**d**).

The energy of the implanted Fe ions used in this study is 72 keV. The ion range is about 33.3 nm, calculated from Stopping and Range of Ion in Matter (SRIM) simulations^10^. The size of NDs used in this experiment is 100 nm in average, therefore, the Fe ions were not energetic enough to penetrate NDs. Hence, we can hypothesize that all implanted Fe ions are in the NDs. High resolution transmission electron microcopy (HRTEM) was used to exam the NDs implanted with Fe after annealing and the result is shown in Fig. 2. The aggregated NDs image is shown in Fig. 2a and that of a high resolution lattice image of a single ND is shown in Fig. 2b. No defects were found in the structure. The Energy-dispersive X-ray spectroscopy (EDX) was used to look at the content of various elements in the ND. The spectrum of an original ND is shown in Fig. 2d. The spectrum of the ND with the image shown in Fig. 2b is shown in Fig. 2c. While before Fe implantation, no Fe signal was found, both K-alpha and K-beta signals of Fe are clearly seen after implant and annealing. Other elements shown in Fig. 2d are probably from surface contamination of the original NDs. But most of them went away after annealing. The Cu signal was from the sample holder and the Si signal was from the Si impurities in the NDs. It was estimated that the Fe content in the ND is around 0.2% in weight by EDX. It was because of the presence of these Fe atoms, the NDs became magnetic.

**Figure 3:**
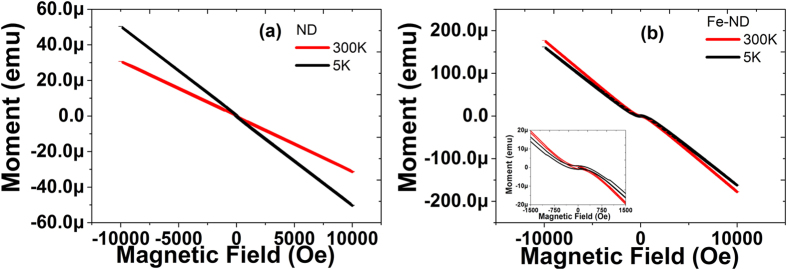
Field dependent magnetization for (**a**) original NDs and (**b**) sorted NDs with dose 3 × 10^15^ cm^−2^ sample at 5 K and 300 K. The original NDs show no hysteresis, it indicates that the original NDs is diamagnetic. The sorted NDs has a clear hysteresis and its saturated moment of is 1.4 × 10^−5^ emu at 5 K and 1.0 × 10^−5^ emu at 300 K, and its coercivity is around 40 Oe. The sorted NDs shows room temperature ferromagnetism. The inset shows the zoom plot of hysteresisfor the sorted NDs.

In this study, the Fe implanted NDs should exhibit magnetic properties, since they were filtered by MACS separator tool first (as described in method). The magnetic characteristics need a comprehensive instrument to measure and is carried out by Quantum Design MPMS3 SQUID-VSM. Figure 3a and b show the NDs’ field dependent magnetization with respect to original NDs and sorted NDs after Fe implant and annealing. No hysteresis loop was observed at 5 K and 300 K in Fig. 3a. It implies that the original NDs are diamagnetic. On the contrary, the Fe implanted sample shows clear hysteresis loop in Fig. 3b. This is an indication that the NDs possess ferromagnetism after Fe ion implantation. The saturated moment was extracted out by subtracting a straight line to remove its diamagnetic background. The extracted saturated moment for the sample, with implanting dose 3 × 10^15^ Fe/cm^2^, is 1 × 10^−5^ emu at 300 K with coercivity of 40 Oe. It is worth to note that the coercivity is much smaller than ordinary macro-scale magnetic material. The small coercivity is a signature of small size ferromagnetic particle due to their small magnetic domain energy^11^. This is in agreement with the Fe implanted NDs, because the size of NDs used in this study is 100 nm.

This ferromagnetic properties can be used on emerging hyperthermia therapy. When magnetic particles are irradiated with an alternating magnetic field (AMF), the energy of the AMF would be transformed to heat by several physical mechanisms, such as hysteresis loses, Neel and Brown relaxation, and viscous loses. It is noteworthy that when the magnetic particles are in small size (nanometer), the Neel and Brown relaxation was shown absorb much more power than hysteresis heating at AMF^12^. The specific absorption rate of magnetic nanoparticles (MNPs) is related to 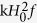, where k is a material constant for a given H and f combination. The Fe implanted NDs behaved small coercivity implies they are suitable nanoparticles for thermal therapy application.

## Conclusion

We have shown that we are able to fabricate NDs with magnetic properties through Fe ion implantation while preserving the original NDs structure with annealing process. The biocompatibility of the original NDs might also be preserved. Then, such implanted NDs can further be developed to adopt the research results found in MNPs and overtake or complement MNPs for biomedical applications usages. The magnetic NDs can be used in various cancer therapies when we need to control the nanoparticles with the external static magnetic field and electro-magnetic radiation. AMF heating is one of the examples. Other applications include enhanced magnetic resonance imaging (MRI) and guiding and localization of the NDs to the needed area for cancer treatment. Although only Fe atoms were implanted in NDs in this work, the technique certainly can include other species that may give NDs with other properties. Adding Boron-10 atoms to NDs by ion implantation will be of particular interest because of the application in BNCT. The technique presented here opens a way for combining the drug delivery capability and the radiation therapy by the same NDs as a carrier agents for cancer treatment. It will give us a new weapon against cancer, which is called theronosic magnetic nanodiamonds.

## Methods

### Sample preparation of Fe implanted NDs

**Figure 4:**
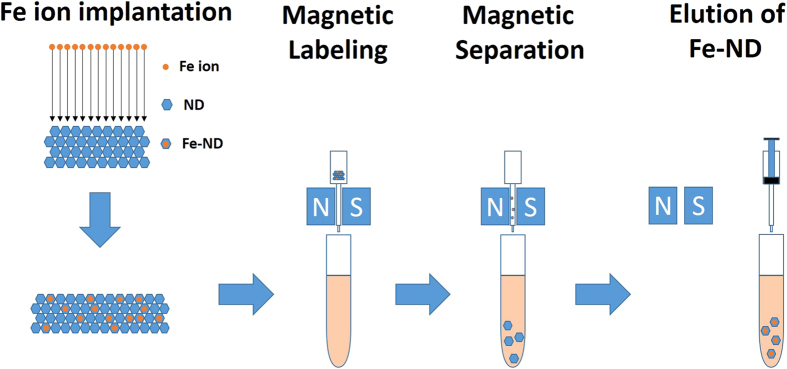
The procedure for Fe ion implantation into NDs and the sorted process with MACS separator tool.

The procedure for Fe ion implantation into NDs is shown in Fig. 4. ND powder (100 nm, Element Six Co.) was first dissolved in distilled deionized water (DD water). The solution was then dropped on an oxidized silicon wafer and dried under a lamp. The Si sample covered by NDs was put in a vacuum chamber for ion implantation.

We use SNICS-II (National Electronics Corp.) ion source to produce Fe ion for implantation with FeP cathode. Fe ions with an energy of 72 KeV at various dosages were implanted into NDs. The Fe ion range in diamond is 33.3 nm with 8.8 nm straggling calculated by SRIM code.

After implantation the samples were annealed at 600 °C for 3 hours in ambient atmosphere for repair the damage of NDs caused during Fe ion implantation. Because the NDs were stacked on the Si wafer with probably many layers, only a portion of the NDs were implanted by Fe ions. The attached NDs were then removed from the Si substrate using an ultrasonic bath of 5 ml DD water. In order to wash out all NDs, we add another 5 ml DD water to flush out the NDs from ultrasonic bath and store the NDs in a tube. The NDs that received Fe ions to become magnetic have to be sorted out using a magnetic filter. We used a magnetic-activated cell sorting tool (MACS Separator, Miltenyi Biotec Co.) as the filter. The Fe doped NDs were attracted by the magnet filter and attached to the adjacent walls when the ND solution passed through the filter. Then the attached magnetic NDs were flushed out by injected 2 ml DD water. Finally the magnetic NDs were collected and stored in a micro-centrifuge tube (eppendorf) with 2 ml DD water.

### Samples preparation for material characteristic analysis

Due to the sorted Fe-implanted NDs within 2 ml DD water stored in the eppendorf is transparency, before we use it for material analysis we used a centrifuge to precipitate the Fe-implanted NDs in the bottom of the eppendorf.

The NDs samples for Raman measurement are prepared by dropping 0.2 ml on 1 × 1 cm^2^ silicon wafer using a pipet. The prepared NDs samples can be observed under optical microscope.

HRTEM/EDX were used to investigate the composition and crystalline structure. The samples for HRTEM/EDS were prepared with pure carbon films. We used pipet to draw 2 micron liter from the bottom of the eppendorf and dropped it directly on the carbon film. The HRTEM/EDS we used is JEOL JEM-2800F equipped with 2 large area 100 mm^2^ silicon drift detectors.

The samples for magnetic properties measurement were prepared with a 5 × 7 mm^2^ silicon wafer dropped with 10 micron liter of the 2 ml sorted NDs from the bottom of the eppendorf. The original NDs we used as control groups for all experiments was 0.02% NDs solution.

## Additional Information

**How to cite this article:** Chen, C. H. *et al*. Fe doped Magnetic Nanodiamonds made by Ion Implantation. *Sci. Rep.* **7**, 41938; doi: 10.1038/srep41938 (2017).

**Publisher's note:** Springer Nature remains neutral with regard to jurisdictional claims in published maps and institutional affiliations.
